# Extranodal natural killer/T-cell lymphoma involving the gastrointestinal tract: analysis of clinical features and outcomes from the Asia Lymphoma study group

**DOI:** 10.1186/1756-8722-6-86

**Published:** 2013-11-16

**Authors:** Seok Jin Kim, Hyun Ae Jung, Shih-Sung Chuang, Huangming Hong, Cheng-Cheng Guo, Junning Cao, Xiao-Nan Hong, Ritsuro Suzuki, Hye Jin Kang, Jong Ho Won, Wee-Joo Chng, Yok-Lam Kwong, Cheolwon Suh, Yu-Qin Song, Jun Zhu, Kevin Tay, Soon Thye Lim, Junji Suzumiya, Tong-Yu Lin, Won Seog Kim

**Affiliations:** 1 Department of Medicine, Division of Hematology and Oncology, Samsung Medical Center, Sungkyunkwan University School of Medicine, Seoul, Korea; 2 Department of Pathology, Chi-Mei Medical Center, Tainan, Taiwan; 3 Department of Medical Oncology, Cancer Hospital, Sun Yat-Sen University, Guangzhou, China; 4 Department of Medical Oncology, Fudan University Shanghai Cancer Center, Shanghai, China; 5 Department of HSCT Data Management, Nagoya University, School of Medicine, Nagoya, Japan; 6 Department of Internal Medicine, Korea Cancer Center Hospital, Seoul, Korea; 7 Department of Internal Medicine, Soon Chun Hyang University Hospital, Seoul, Korea; 8 Department of Hematology–Oncology, National University Cancer Institute of Singapore, National University Health System, Singapore, Republic of Singapore; 9 Department of Medicine, Queen Mary Hospital, Hong Kong, China; 10 Department of Oncology, Asan Medical Center, University of Ulsan College of Medicine, Seoul, Korea; 11 Lymphoma Department, Peking University Cancer Hospital & Institute, Beijing, China; 12 Lymphoma Division, National Cancer Center, Singapore, Singapore; 13 Cancer Center, Faculty of Medicine, Shimane University, Izumo, Shimane, Japan

**Keywords:** Extranodal NK/T-cell lymphoma, Gastrointestinal tract, Prognosis

## Abstract

**Background:**

The gastrointestinal (GI) tract is one of the most common extranasal sites in extranodal NK/T-cell lymphoma (ENKTL). However, data regarding ENKTL involving the GI tract are relatively scarce. Thus, we performed a multicenter, multinational retrospective study to analyze clinical features and treatment outcomes of ENKTL involving the GI tract.

**Patients and methods:**

Patients with ENKTL involving the GI tract diagnosed in twelve participating centers between 1991 and 2012 were retrospectively analyzed from five Asian countries.

**Results:**

The analysis of 81 patients with ENKTL involving the GI tract revealed that more than 60% of patients presented as advanced disease with B symptoms. 55 patients (68%) had GI manifestations including abdominal pain (n = 26, 32%), GI tract bleeding (n = 17, 21%) and bowel perforation (n = 12, 15%). The most common GI site was the small intestine, including the jejunum and ileum (n = 57, 70.3%). There were 34 patients (42%) who received systemic chemotherapy while 33 patients (41%) underwent surgery plus chemotherapy. However, 35 patients (43%) died due to disease progression, and treatment-related mortality including sepsis occurred in 17 patients (21%). Thus, the median overall survival was 7.8 months (95% Confidence interval: 3.9 – 11.7 months). Patients who could undergo surgery plus chemotherapy showed a trend of better survival than those treated with chemotherapy alone.

**Conclusion:**

Overall, the data indicated that ENKTL involving the GI tract has a dismal prognosis despite active treatment including chemotherapy and surgery. Thus, more effective treatment strategies are required for this disease entity.

## Background

Extranodal NK/T cell lymphoma (ENKTL) is known to cause localized disease involving midline facial structures such as the nasal cavity and nasopharynx [1]. However, the occurrence of ENKTL outside the nasal areas such as the skin, liver, soft tissue and gastrointestinal (GI) tract is also common. Furthermore, cases involving extranasal areas frequently present as more advanced disease and have poorer prognoses than nasal cases. Accordingly, a recent retrospective study showed that the worst treatment outcomes were associated with skin and soft tissue involvement, especially in cases of advanced disease [2]. However, data regarding ENKTL involving the GI tract are relatively scarce [3], although the GI tract is the most common site of extranodal organ involvement in patients with non-Hodgkin lymphoma (NHL) [4,5]. A previous retrospective analysis of 581 patients with intestinal NHL reported a frequency of 3.1% of ENKTL (n = 18) and poorer prognosis than B-cell lymphomas [6]. In the study, however, because of the small number of ENKTL patients, the results might not be representative. Furthermore, the treatment outcomes of various treatment modalities for patients with GI tract ENKTL have hitherto not been compared. The role of surgery in GI tract ENKTL is also undefined, although it appears to be an important modality in the management of localized intestinal B-cell lymphomas [7]. As a result, there is still no established treatment strategy for ENKTL involving the GI tract.

In this study, we conducted a multicenter retrospective analysis of a cohort of Asian patients with GI tract ENKTL, in order to define the clinicopathologic features, and the contribution of different treatment modalities to treatment outcome.

## Materials and methods

### Patients

Patients with a diagnosis of ENKTL involving the GI tract diagnosed in twelve participating centers were retrospectively analyzed. Patients were accrued during two periods, from 1991 – 2000, the pathologic diagnosis was based on the Revised European-American Lymphoma (REAL) classification. From 2001 – 2012, the diagnosis was based on World Health Organization (WHO) classification criteria. Pathologic data of patients diagnosed according to the REAL classification were reviewed to ensure that they fully fulfilled the WHO criteria. The GI tract was considered as primarily involved if patients presented initially with GI symptoms or complications. Patients who presented with nasal lesions were also analyzed if the involvement of GI tract was found at the staging work-up. Tumor locations were determined using imaging findings, such as computerized tomography (CT), or surgical findings if surgical resection was performed. The Ann Arbor staging system was used for staging investigations included complete blood counts, serum biochemistry, serum lactate dehydrogenase (LDH), bone marrow aspiration and trephine biopsy, nasal endoscopic examination and computed tomographic (CT) scanning of the involved organ(s), chest and abdomen.

### Clinical data analysis

Clinicopathologic data including demographics, primary presentation such as GI manifestations, stage, Eastern Cooperative Oncology Group (ECOG) performance status, serum LDH, the presence of B symptoms, and tumor location were analyzed. Treatment modalities, including surgery and chemotherapy (first-line regimens) were also analyzed. Potential risk factors were analyzed including the International Prognostic Index (IPI) as well as the NK prognostic index (NKPI, as determined by presence of B symptoms, stage III/IV, elevated serum LDH, involvement of regional lymph nodes) [8]. This study was approved by the Institute Review Board of Samsung Medical Center (No. 2013-07-079).

### Statistical analysis

Overall survival (OS) was calculated from the date of diagnosis to the date of the last follow-up visit or death from any cause. Survival was estimated using Kaplan–Meier curves and compared by the log-rank test. The follow-up duration was calculated by the method of Kaplan-Meier estimate of potential follow-up as previously reported [9].The Cox proportional hazard regression model was used in multivariate analyses to identify prognostic factors. Two-sided *P*-values < 0.05 were considered significant.

## Results

### Patient characteristics

**Figure 1:**
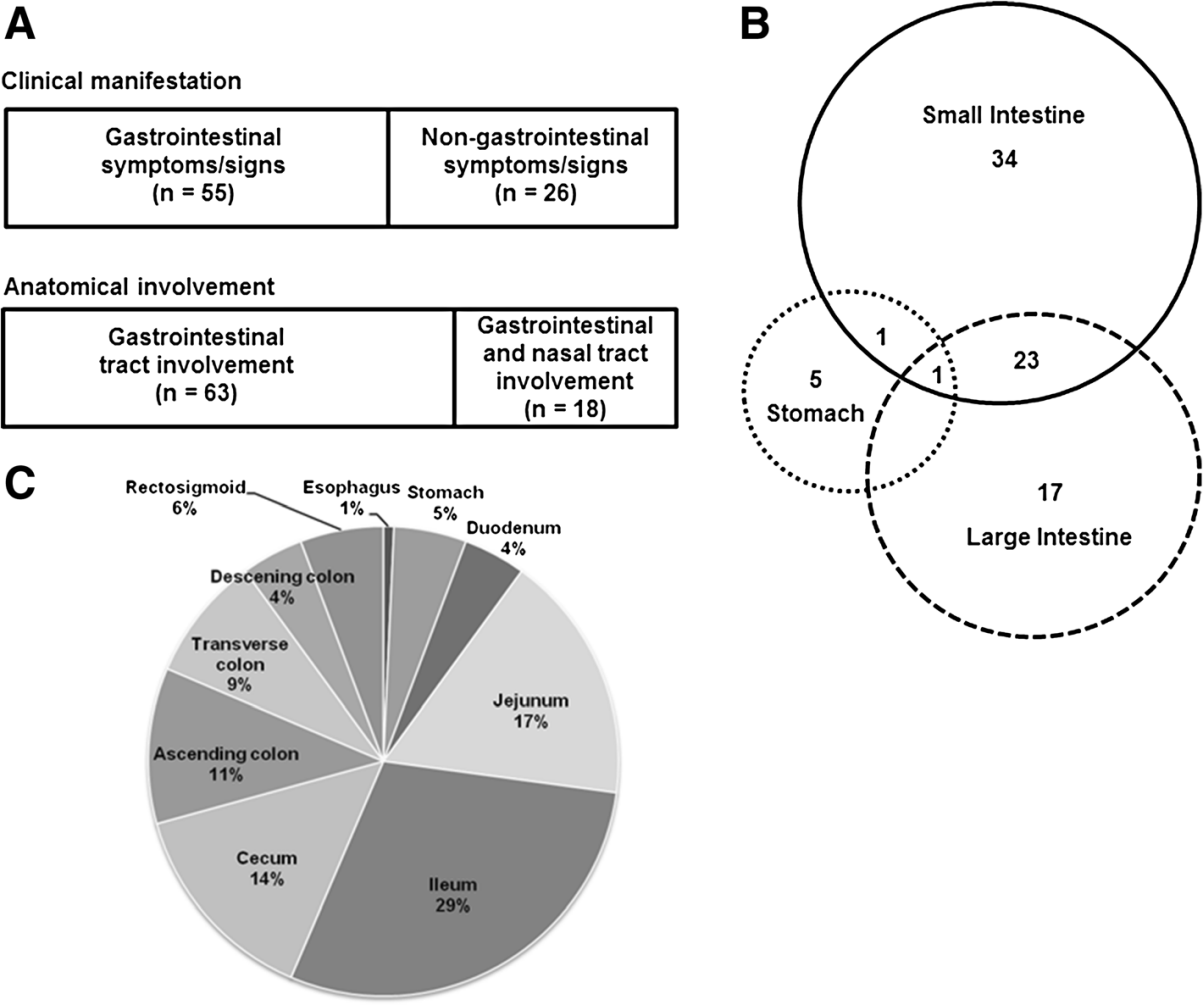
**Clinical manifestation and gastrointestinal tract involvement. (A)** Comparison of clinical manifestation and anatomic involvement **(B)** Pattern of gastrointestinal tract involvement. **(C)** Frequency of involvement of each part of the GI tract by extranodal NK/T-cell lymphoma from the esophagus to the recto-sigmoid colon (The percentage of each part of GI tract = the number of patients involving each part/total number of involved parts in 81 patients × 100).

Eighty-one patients were included in this retrospective analysis. The median age of patients at diagnosis (45 years, range: 17–79 years), and male predominance (69%, 56/81) were similar to the overall characteristics reported for ENKTL [10]. However, approximately 65% of patients had two or more sites of extranodal involvement (Table 1). Thus, the proportion of advanced disease was over 60%, and B symptoms were also frequently observed (63%, 51/81). As a result, risk stratification based on the NKPI showed that > 50% of patients belonged to the high-risk category of group 4. In contrast, the IPI showed only 11 patients were grouped as high-risk, because the majority of patients were younger than 60 years of age (Table 1). GI manifestations were the main symptom at diagnosis in 55 patients (68%) and included abdominal pain (n = 26, 32%), GI tract bleeding (n = 17, 21%) and bowel perforation (n = 12, 15%). Thus, a substantial number of patients underwent surgery to remove their primary mass for diagnostic as well as therapeutic purposes. The other 26 patients (32%) presented as non-GI manifestations including nasal obstruction (n = 6) or systemic symptoms (n = 20) such as fever and night sweat. During the evaluation of these 26 patients including endoscopic examination of nasal cavity and CT scans of neck, chest and abdomen-pelvis, the simultaneous involvement of nasal cavity or nasopharynx as well as GI tract was found in 18 patients (22%). Thus, these 18 patients were pathologically diagnosed with ENKTL by biopsy of nasal areas, and the involvement of GI tract was documented in abdominal CT scan and/or positron emission tomography (PET)/CT scan. The remaining eight patients out of 26 patients presented with non-GI manifestation also underwent surgery to remove mass of GI tract for pathologic diagnosis (Figure 1A).

**Table 1:** Patient characteristics

**Characteristics**		**Number (%)**
Sex	Male	56 (69)
	Female	25 (31)
Age (years)	≤ 60	67 (83)
	> 60	14 (17)
Performance status	ECOG 0/1	54 (67)
	ECOG ≥ 2	25 (31)
	Missing	2 (2)
Extranodal involvement	< 2	29 (36)
	≥ 2	52 (64)
Serum LDH	Normal	38 (47)
	Increased	40 (49)
	Missing	3 (4)
Ann Arbor stage	I/II	18 (22)/11 (13)
	III/IV	7 (9)/45 (56)
B symptoms	Absence	30 (37)
	Presence	51 (63)
Bone marrow invasion	Absent	68 (84)
	Present	7 (9)
	Not evaluated	6 (7)
NK Prognostic Index	Group 1/2	3 (4)/12 (15)
	Group 3/4	21 (26)/42 (52)
	Missing	3 (4)
International Prognostic Index	Low/Low-intermediate	22 (27)/19 (24)
	High-intermediate/High	26 (32)/11 (13)
	Missing	3 (4)
Clinical presentation	GI manifestation	55 (68)
	Non-GI manifestation	26 (32)
Nasal involvement	Absence	63 (78)
	Presence	18 (22)

### Pattern of GI tract involvement

ENKTL dominantly involved the small and large intestine (n = 58, 72%) rather than stomach. The small intestine was the most commonly involved site as 34 patients (42%) had small intestinal disease only while 23 patients (28%) had disease involving both the small and large intestine. One patient had disease in the stomach, duodenum, and jejunum, and the other patient presented with disease in the stomach, duodenum and descending colon (Figure 1B). Given a patient could involve multiple involvement of GI tract such as small and large intestine, the absolute number of involved parts of GI tract in this study was higher than the number of patients. Hence, when we analyzed the percentage of involvement through whole GI tract, the most commonly involved part was ileum (29%). Jejunum and cecum were 17% and 14%, respectively. Thus, these three parts of GI tract accounted for approximately 60% of GI site involvement (Figure 1C). In addition, 17 patients (21%) had intra-abdominal involvement in sites including the liver, pancreas, adrenal glands, and peritoneum.

### Treatment

**Figure 2:**
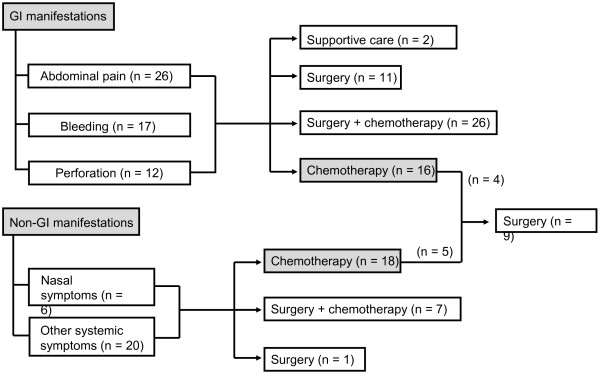
Summary of treatment approaches.

Out of the 55 patients presenting with GI manifestations, 37 patients first underwent resection of their primary mass lesion mainly for complications such as bleeding or perforation (Figure 2). All but one patients with bowel perforation underwent surgery. One patient presenting as abdominal pain and the other patient pretesting as perforation received only supportive care because they could not undergo surgery owing to their poor health status s (Figure 2). After surgery, 26 patients received systemic chemotherapy, whereas 11 patients did not. Sixteen patients who had widespread disease including GI tract and other sites received systemic chemotherapy (Figure 2). Among the 26 patients presenting without GI manifestations, 18 received systemic chemotherapy while 8 underwent surgery first. Therefore, the initial treatment for these patients included chemotherapy alone (n = 34, 42%), surgery plus chemotherapy (n = 33, 41%), and surgery alone (n = 12, 15%). CHOP (cyclophosphamide 750 mg/m^2^ on day1, doxorubicin 50 mg/m^2^ on day 1, vincristine 1.4 mg/m^2^ on day 1, and prednisone 100 mg on days 1–5) or CHOP-like chemotherapy regimens were used in 37 patients whereas non-anthracycline-based or intensified chemotherapy such as SMILE (Methotrexate 2 g/m^2^ on day 1, ifosfamide 1500 mg/m^2^ on days 2–4, etoposide 100 mg/m^2^ on days 2–4, dexamethasone 40 mg on days 2 – 4, and *Escherichia coli* L-asparaginase 6,000 U/m^2^ on days 8, 10, 12, 14, 16, 18, 20, n = 17), EPOCH (etoposide 50 mg/m^2^ on days 1–4, doxorubicin 10 mg/m^2^ on days 1–4, vincristine 0.4 mg/m^2^ on days 1–4, cyclophosphamide 750 mg/m^2^ on day 5, and prednisolone 60 mg/m^2^ on days 1–5, n = 5), VIPD (etoposide 100 mg/m^2^ on days 1–3, ifosfamide 1500 mg/m^2^ on days 1–3, cisplatin 33 mg/m^2^ on days 1–3, and dexamethasone 40 mg on days 1 – 4, n = 2), ESHAP (etoposide 40 mg/m^2^ on days 1–4, methylprednisolone 500 mg on days 1–4, cisplatin 25 mg/m^2^ on days 1–4, and cytarabine 2 g/m^2^ on day 5, n = 1), gemcitabine-containing chemotherapy (n = 2), and others (n = 3) were used in 30 patients. However, among the 34 patients treated with systemic chemotherapy, 9 patients underwent surgery due to GI complications during chemotherapy (Figure 2) including bowel perforation (n = 4), GI bleeding (n = 2), and GI obstruction (n = 3). Autologous (n = 3) or allogeneic hematopoietic stem cell transplantation (HSCT, n = 5) was done in eight patients as a consolidation treatment after chemotherapy.

### Treatment outcomes

**Figure 3:**
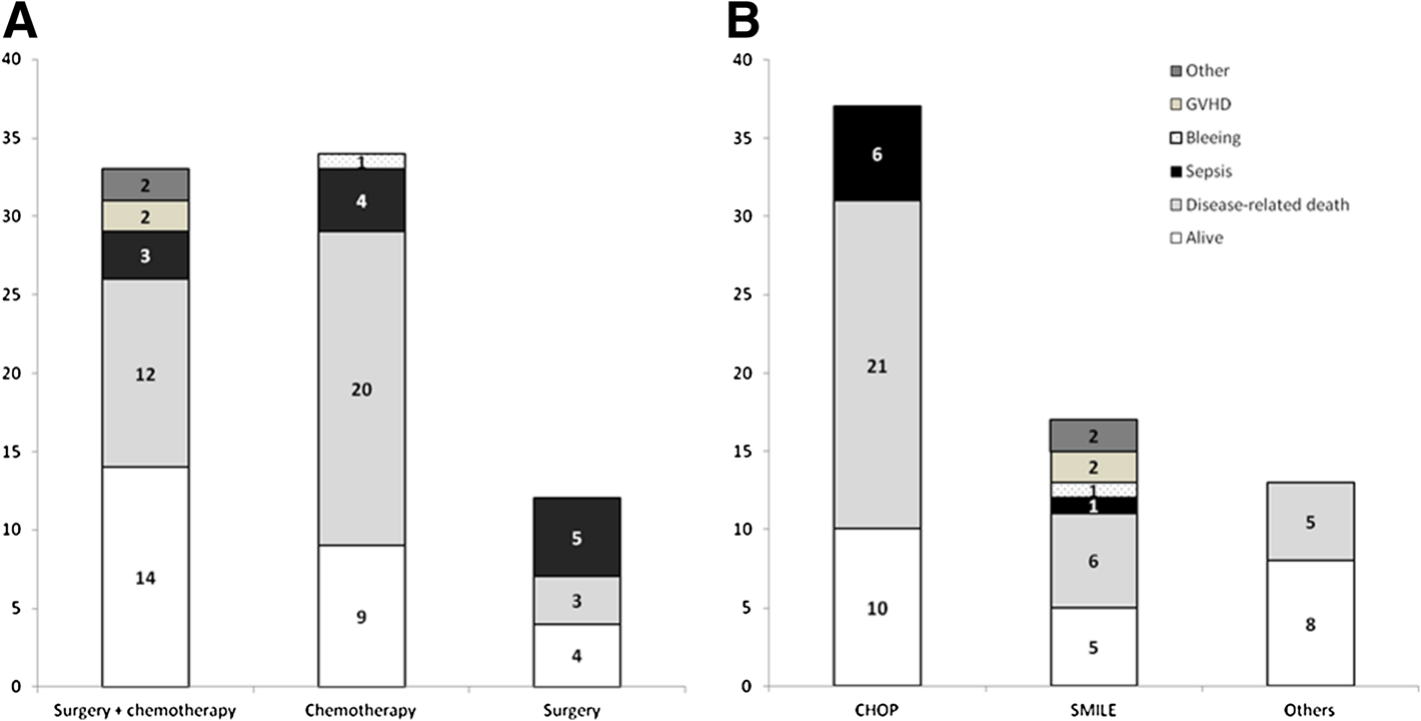
**Comparison of treatment outcomes based on treatment modality and chemotherapy regimen. (A)** Treatment outcomes for patients treated with chemotherapy or surgery or both. **(B)** There is no significant difference of death causes according to the type of chemotherapy regimens.

**Figure 4:**
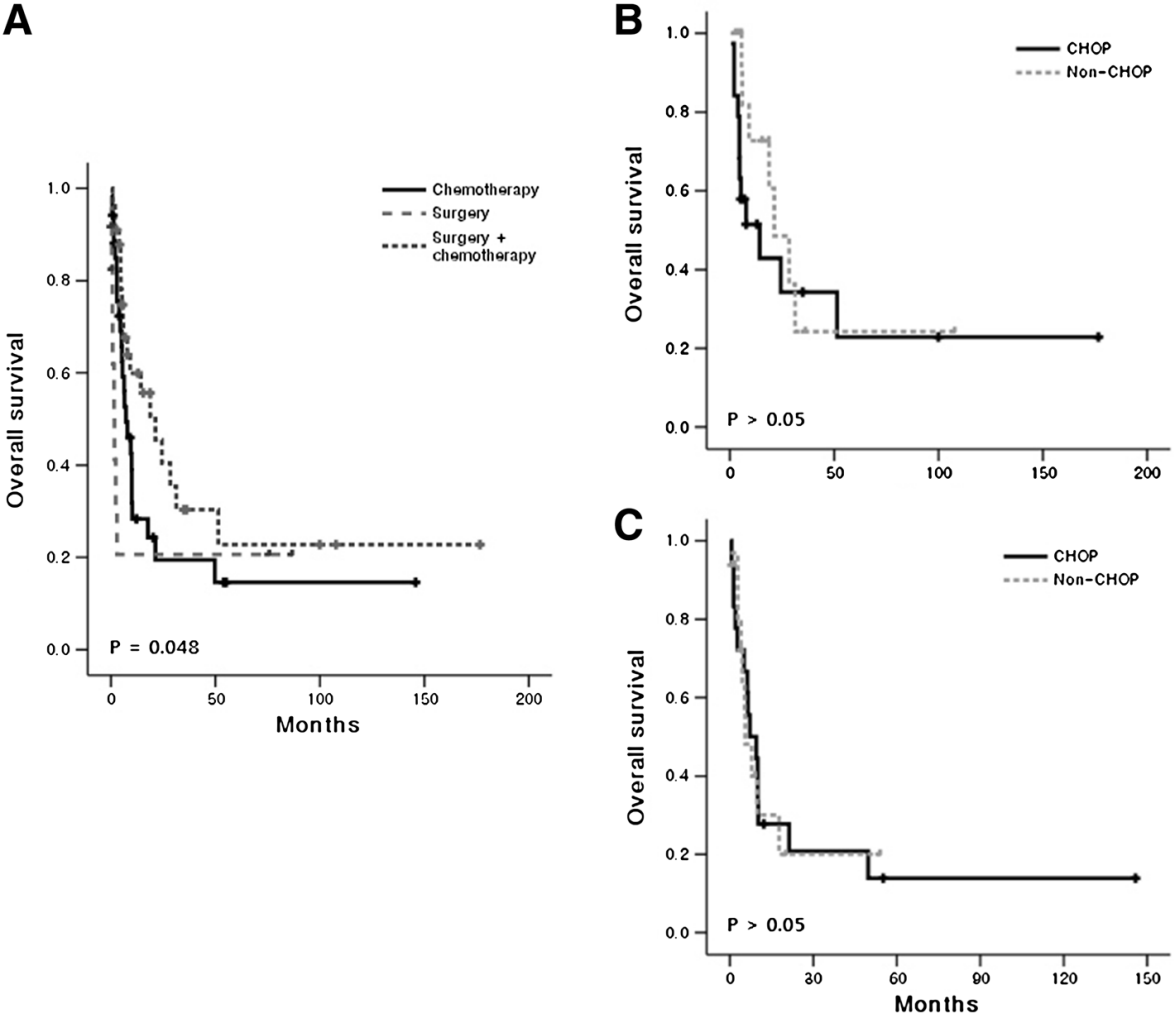
**Analysis of survival outcomes based on treatments. (A)** The comparison of overall survival shows better survival of patients treated with surgery plus chemotherapy with marginal significance compared to chemotherapy alone. **(B)** The comparison of overall survival according to the type of chemotherapy regimen, CHOP versus non-anthracycline based chemotherapy (non-CHOP) shows no difference in the surgery plus chemotherapy group. **(C)** In the chemotherapy group, there is no difference between CHOP and non-CHOP regimens.

With the median potential follow-up of 54.0 months (95% Confidence interval (CI): 31.6 – 76.4 months), the median OS was only 7.8 months (95% CI: 3.9 – 11.7 months). A total of 54 patients died, including two patients who did not receive any curative treatment. Among the 79 patients who received surgery or chemotherapy or both, 35 patients died due to disease progression while non-disease-related deaths were found in 17 patients (Figure 3A). Sepsis accounted for 70% of non-disease-related death (12/17), with seven patients dying from sepsis after chemotherapy. In patients treated with surgery plus chemotherapy, two patients died due to graft-versus host disease (GVHD) after allogeneic HSCT, and the remaining two other deaths included surgical complication-related death and suicide (Figure 3A). The comparison of outcomes according to chemotherapy regimens showed no significant difference in the cause of deaths among patients who received CHOP or SMILE regardless of surgery although the proportion of disease-related death was lower in patients who received SMILE (35%, 6/17) than CHOP (57%, 21/37, Figure 3B). The median OS of patients treated with surgery plus chemotherapy was 21.3 months (95% CI: 8.2 – 34.4 months) whereas the median OS of chemotherapy or surgery alone was 7.2 (95% CI: 3.0 – 11.4 months) and 1.7 months (95% CI: 0.36 – 3.0 months), respectively. Thus, surgery followed by chemotherapy showed a tendency toward better outcomes compared with chemotherapy (Figure 4A). However, these results might be related to the fact that patients who received chemotherapy had more advanced disease and unfavorable characteristics compared with patients who underwent surgery plus chemotherapy (Table 2). When we compared the OS according to chemotherapy regimens, patients who received non-anthracycline-based or intensified regimens did not show a significant survival difference compared with patients who received CHOP or CHOP-like regimens regardless of surgery (Figure 4B, C). The comparison of CHOP with SMILE also showed no survival difference (data not shown).

**Table 2:** Comparison of characteristics based on treatment

**Characteristics**	**Surgery + Chemotherapy**	**Chemotherapy**	***P***
Age (years)			
≤ 60	25 (76)	30 (88)	0.13
> 60	8 (24)	4 (12)	
Performance status			
ECOG 0/1	27 (84)	20 (59)	0.03
ECOG ≥ 2	5 (16)	14 (41)	
Extranodal involvement			
< 2	15 (45)	7 (21)	0.04
≤ 2	18 (55)	27 (79)	
Serum LDH			
Normal	17 (52)	15 (44)	0.62
Increased	15 (45)	19 (56)	
Missing	1 (3)		
Ann Arbor stage			
I/II	15 (45)	5 (15)	< 0.01
III/IV	18 (55)	29 (85)	
B symptoms			
Absence	15 (45)	9 (27)	0.13
Presence	18 (55)	25 (73)	
Bone marrow invasion			
Absence	29 (88)	29 (85)	0.20
Presence	2 (6)	5 (15)	
Not evaluated	2 (6)	0 (0)	
Nasal involvement			
Absence	30 (91)	19 (56)	< 0.01
Presence	3 (9)	15 (44)	

### Prognostic factors

**Figure 5:**
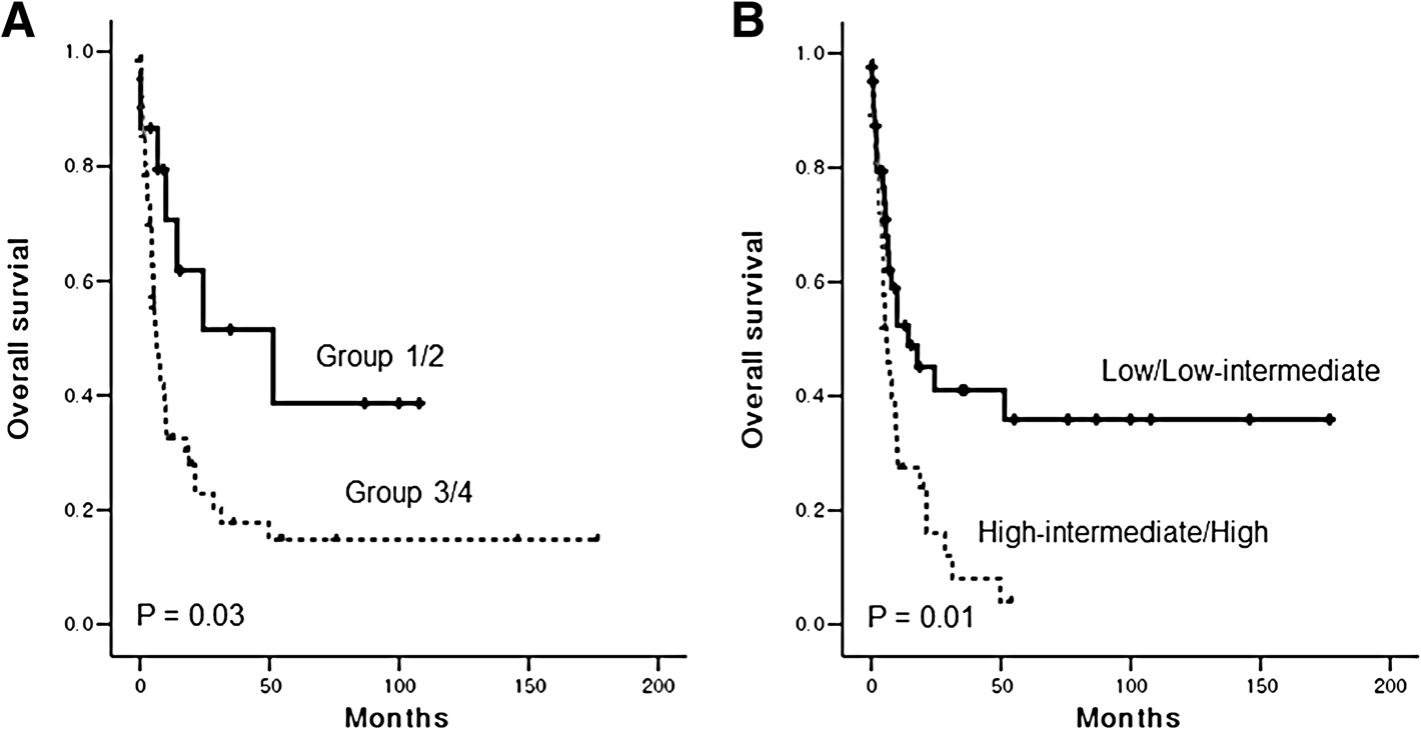
**Analysis of survival outcomes based on prognostic models. (A)** Patients belonged to groups 1 and 2 of the NKPI showed better overall survival than groups 3 and 4 of the NKPI **(B)** High or high-intermediate risk group of the IPI showed worse overall survival than patients with low or low-intermediate risk of the IPI.

The OS was not significantly different on univariate analysis according to age > 60 years, serum LDH elevation, stage III/IV, B symptoms, location of GI tract involvement, nasal involvement, or bone marrow invasion (*P* > 0.05). Only extranodal involvement and performance status were significantly associated with worse OS (*P* < 0.05). Thus, patients who had involvement at two or more than two extranodal sites and poor performance status showed inferior OS. However, multivariate analyses failed to show an independent prognostic factor for ENKTL involving the GI tract. The prognostic model, IPI and NKPI showed a significant association with poor OS (Figure 5A, B).

## Discussion

This is a multinational, multicenter study analyzing to date the largest number of patients with ENKTL involving the GI tract. Although there may have been selection bias owing to the retrospective nature of the study, our results reflect the clinical features and treatment outcomes of GI ENKTL. In this study, the majority of patients presented with advanced disease and the simultaneous involvement of other extranodal sites such as nasal tract. Furthermore, multiple areas of involvement throughout the GI tract were also frequent. Therefore, detailed determination of the extent of disease is necessary in patients with ENKTL who present with GI tract disease. Our study showed a predominant involvement of the small intestine, especially the ileum and jejunum. This pattern of involvement was different from B-cell lymphomas, which more commonly involve the stomach, terminal ileum and cecum. This frequent involvement of the jejunum and ileum by ENKTL is similar to that of peripheral T-cell lymphoma [6]. As ENKTL patients showed frequent involvement of the jejunum and ileum as well as GI complications such as bleeding and perforation, surgical resection of the primary mass was inevitable for diagnosis and treatment in the majority of our cases. After surgery, systemic chemotherapy was originally planned in all patients due to the nature of the systemic disease. However 12 patients only underwent surgery without any adjuvant treatment in this study. The predominant reason for not administering subsequent chemotherapy was due to general poor-health post-operatively. As a result, patients who underwent surgery alone showed the extremely short survival duration (median OS: 1.7 months). On the other hand, the OS of patients who underwent surgery followed by chemotherapy was better than patients who were initially treated with chemotherapy, although the statistical significance was marginal (Figure 4A). Given the fact that stage I/II disease and good performance status were more common in patients who received surgery and adjuvant chemotherapy (Table 2), the better outcome of surgery plus chemotherapy than that of chemotherapy alone might be associated with tumor burden and performance status. Nevertheless, our study results suggested surgery plus chemotherapy might be beneficial for patients who were eligible for surgery such as localized disease and good performance status.

The outcome of patients who received chemotherapy only was extremely poor, with the majority of patients dying due to disease progression (20/34, 59%, Figure 3A), as compared with 12 patients dying from disease progression in the surgery plus chemotherapy group (12/33, 36%, Figure 3A). Although patients who received chemotherapy had more advanced disease (*P* < 0.01, Table 2), our results imply the efficacy of chemotherapy is still not sufficient to overcome this disease entity. Considering inferior efficacy of CHOP regimen for ENKTL due to frequent occurrence of resistance to doxorubicin, we compared the cause of death according to the type of chemotherapy regimen. Although there was no significant difference in the cause of deaths among patients who received CHOP or SMILE regardless of surgery, it seemed to be that the proportion of disease-related death was lower in patients who received SMILE (35%, 6/17) than CHOP (57%, 21/37, Figure 3B). However, the outcome of non-anthracycline-based chemotherapy such as SMILE failed to show a survival benefit compared with the CHOP regimen (Figure 4B, C), because of high proportion of treatment related mortality [11,12]. These results imply that intensified regimens still might be insufficient to control this disease entity and produce unacceptable toxicity leading to treatment-related morbidity and mortality. The inferior outcome of our patients might also be related to the fact that a substantial number of patients died due to treatment-related complications, especially sepsis (Figure 3A). Furthermore, nine patients had to undergo surgery during chemotherapy due to perforation or bleeding. This frequent occurrence of treatment-related complications might be associated with the predominant intestinal involvement by ENKTL and the aggressive nature of this disease. Thus, much more attention is required in monitoring of the occurrence of complications during treatment, and active preventive approaches including antibacterial prophylaxis might be helpful for improving treatment outcomes in these patients. There was no unfavorable prognostic factor specific to patients with ENKTL involving the GI tract. Univariate analysis showed that there was an association between tumor burden and performance status and poor OS as is seen with other NHL.

In conclusion, ENKTL with GI tract involvement showed predominant involvement of the small intestine. The prognosis was extremely poor despite active treatment including chemotherapy and surgery. In addition, treatment-related mortality was also relatively high and included cases of sepsis. Thus, a more effective treatment strategy should be developed for this rare but fatal disease entity.

## Competing interests

The authors declare that they have no competing interests.

## Authors’ contributions

SJK and WSK designed the research, and analyzed the data. SJK and HAJ wrote the paper. S-SC, HH, C-CG, Y-QS, KT, X-NH, JC, JZ, RS, HJK, JHW, JS, T-YL, W-JC, STL, CS, Y-LK performed the research, and contributed study materials. WSK, Y-LK, and S-SC edited the manuscript. All authors read and approved the final manuscript. All authors read and approved the final manuscript.
